# Safe Hugs in Palliative Care

**DOI:** 10.5152/TJAR.2022.21308

**Published:** 2022-08-01

**Authors:** Aykut Sarıtaş, Uğur Uzun, Pelin Uzun Sarıtaş

**Affiliations:** 1 Department of Anaesthesiology and Reanimation and Palliative Care, Health Sciences University İzmir Tepecik Training and Research Hospital, İzmir, Turkey

**Keywords:** Hospice, mnemonics, nutrition, pain, palliative care

## Abstract

Mnemonics are word formulations that aid physicians in recalling instances hidden among typical applications and may be neglected due to workload. Mnemonic abbreviations that benefit not only physicians but the entire care team are widely used throughout the world. Given that palliative care is the work of a multidisciplinary team, these mnemonics become even more significant. The aim of this study is to introduce the acronym “SAFE HUGS IN PC” (Sleep patterns, Analgesia, Feeding, Environment, Hospital Discharge-Home Care, Ulcer, Gastrointestinal, Social Support-Spiritual, Infection, Need of Religion, Physiotherapy-Psychotherapy, Goals of Care), which we believe will meet the requirements in the palliative care. The following databases were searched: CINAHL, Cochrane, Embase, MEDLINE, and Pubmed for studies exploring experiences of palliative care. With this mnemonic, which we use in our own clinic, patients’ and patients’ relatives’ satisfaction and end-of-life quality have increased. We believe this simple mnemonic will encourage teamwork and help improve the quality of life on palliative care.

**Main Points**

- Palliative care (PC) refers to a multidisciplinary process. It is essential that daily support, treatment and follow-ups are carried out sensitively with a multidisciplinary approach.
- The use of mnemonics to provide quality care has a proven benefit.
- Mnemonic abbreviations that benefit not only physicians but the entire care team are widely used throughout the world.
- The aim of this study is to introduce the acronym ‘’SAFE HUGS IN PC’’ which we believe will meet the requirements in the PC.

## Introduction

The number of people living with life-threatening chronic diseases is increasing in recent years as life expectancy has increased. Therefore, the popularity and necessity of palliative care (PC) are gaining importance day by day. Palliative care is a type of care that is not only provided to oncology patients but also covers the service provided for all kinds of life-threatening diseases. Palliative care is defined as an approach that should be applied in the early stages of a disease, with active treatment beginning with the diagnosis of a life-threatening disease and other treatments aimed at prolonging life and continuing with the support of the patient’s relatives after death or the surviving patient’s rehabilitation process.^1,2^ In the bow-tie model, Hawley^3^ stated that the patient may not always die at the end of PC and that the rehabilitation process can be maintained, contrary to the traditional understanding.

Palliative care aims to improve the quality of life for patients and their families by reducing pain and other symptoms related to the disease, assist in the treatment process, and provide moral and psychological support to both the patients and their relatives.^4^ Palliative care refers to a multidisciplinary process. Therefore, it is essential to carry out daily support, treatment, and follow-ups with precision. We believe that some mnemonics frequently used in intensive care units will be handy and crucial in PC both to improve patient care quality and prevent potential errors. Vincent^5^ published the FAST HUGS mnemonic to identify and control critical aspects in the overall care of critically ill patients admitted to the intensive care unit. Following that, FAST HUGS modifications and many similar abbreviations were published.^6-8^ It is concluded that with these mnemonic abbreviations, practical applications become more significantly easier, complications are reduced, and professional medical care quality and treatment success are increased.

We believe that arranging these proven mnemonics according to the purpose of PC and using them as checklists will contribute significantly to the improvement of patient care quality and strengthen communication with patients and their relatives to determine care needs and not to ignore the necessary care goals.

These checklist abbreviations, which will be created in PC services, will enable both the physicians who organize the treatments and the assistant health team who perform the follow-up and treatment to do their work correctly and accurately.

We introduced and recommended a PC-specific version of a checklist (Sleep patterns, Analgesia, Feeding, Environment, Hospital Discharge-Home Care, Ulcer, Gastrointestinal, Social Support-Spiritual, Infection, Need of Religion, Physiotherapy-Psychotherapy, Goals of Care [SAFE HUGS IN PC]) equivalent to the mnemonics FAST HUGS and that has proven effective in intensive care units with its reminding power.

### Clinical and Research Consequences

To highlight some crucial aspects in the daily general care of all PC patients, we want to propose the concept of SAFE HUGS IN PC (Table 1) as a helpful and straightforward mnemonic. This mnemonic helps to include all palliative physicians, nurses, physiotherapists, psychiatrists, nutrition teams, and chaplains in PC. The aim of introducing the mnemonic is to prevent PC team members from being mentally overwhelmed, eliminate probable care defects, improve care quality, promote communication with patients and their families, and create an effective treatment environment.

In our own clinic, we use the SAFE HUGS IN PC mnemonic in order to implement a memorable method that includes all the necessary parametres in the follow-up of our patients, suitable for practical application. We put forth the importance of this mnemonic and the content of its subheadings as follows:

**S for Sleep Patterns: **Sleep disturbance is a term used to describe symptoms of perceived or actual sleep pattern change.^9^ Sleep disturbance is stated as less than 7 hours of sleep, sleep latency of more than 20 minutes, wake after sleep onset (WASO) of more than 10%, daytime napping of more than 2 hours, and a sleep efficiency of less than 80%.^10^ Sleep disturbance is a significant problem, especially for palliative patients with a life-threatening disease.

Inadequate or frequently interrupted sleep state may contribute to daytime fatigue and weakness, cognitive decline, agitation, increase in the potential for depression and delirium, reluctance to physiotherapy, increase in symptoms including pain, nutritional deficiencies, immune deficiency, treatment rejection, and deterioration in the quality of life.^11,12^ Sleep disturbances can also affect caregivers, increasing their workload and leading to burnout syndrome.^13^ Studies have shown that 42%-95% of caregivers of patients with terminal cancer have mild to severe sleep disturbances and may persist not only during the provision of care and attention but also after the patient’s death.^14,15^ The deterioration of the quality of life of caregivers seriously affects the care and support provided, causing an unavoidable avalanche effect. The effect of this avalanche becomes not only the patient’s problem but also the problem of the entire palliative team, including nurses, social workers, physiotherapists, and psychotherapists. The main thing is to take appropriate measures to prevent this avalanche effect before it occurs. Therefore, it is crucial to control the sleep pattern at least once a day in the daily evaluation of palliative patients. A malfunction that occurs in a single chain will render the wheel unworkable in scenarios connected like a chain. That is how critical sleep is for the wheel to turn in palliative patients.

The treatment approach for a sleep disorder is usually pharmacological (Table 2). However, non-pharmacological treatments, such as music, have been demonstrated to be more effective than traditional methods in several studies.^16,17^ The most effective treatment is to use daily checklists to identify predisposing factors and to prevent sleep disturbance before it arises. Checklist control with mnemonics can play an active role in identifying and preventing sleep disorders. The pain factor, which is one of the most important predisposing factors leading to sleep disorder, is stated below, together with 5 components of A as the second letter of the mnemonic.^18,19^

**A for Analgesia (5 components of A): **The greatest fear among individuals with a life-threatening illness is pain. In PC, effective pain management is crucial, which may act as a predisposing factor for many symptoms. Inadequate pain management has several negative consequences, including sleep disturbances, malnutrition, immunodeficiency, delirium, depression, physiological inadequacy, and a detrimental impact on the quality of life of patients and their families.

Although it has been stated that 75%-90% of end-of-life pain can be effectively managed, pain rates are high even in people receiving PC.^20-22^ Inadequate pain management may be due to many factors as follows:^21,23^

- clinician’s failure to assess or care about pain,
- believing that pain is a usual symptom in patients in need of PC (“That much pain is normal” mentality),
- pain to be overlooked,
- opiophobia of the clinician,
- belief that after early pain management, there will be no option for future pain management,
- concern about unwanted side effects of pain relievers,
- patient’s reluctance to express pain (desire to be a good patient, belief that increasing pain worsens illness, and willingness to deny it).

Pain should be evaluated during the day. Among the scoring systems to evaluate this, the most accepted are the Visual Analog Scale, Verbal Rating Scale, and Wong–Baker FACES Pain Rating Scale. Mnemonics contribute to communication with patients and caregivers, increasing pain awareness and the success of appropriate pain management.

Effective pain management involves a multidimensional approach that includes personalized pharmacological and non-pharmacological interventions according to the patient’s specific situation.^24^ In the pharmacological management of pain, the use of non-opioid analgesics such as acetylsalicylic acid and acetaminophen, nonsteroidal anti-inflammatories, and opioids can be gradual and combined, or it can be supported by additional adjuvant methods (Table 3).^25-29^

Non-pharmacological methods are therapeutic complements to pain-relieving drugs that decrease the need for greater doses and the incidence of possible side effects. These methods can help reduce pain and distress. Approaches include palliative radiotherapy, complementary/alternative methods, focused relaxation and breathing, acupuncture, and cognitive/behavioural techniques.^30,31^

Agitation and anxiety, with or without pain, are common in PC patients. Anxiety is usually a feeling of fear, worry, and restlessness. Frequently, the patient is unable to pinpoint the source of the anxiety and feels uncertain about the future. The main treatment of anxiety is non-pharmacological approaches.^32^ In cases of severe anxiety, the addition of pharmacological treatment may be helpful. Antidepressant is used both as an adjuvant in the treatment of pain and in the prevention of anxiety-depression in PC patients. It can cause unrelieved pain, agitation, and anxiety. The use, necessity, and dose adjustment of antidepressants and anti-convulsions can be easily determined with these symptoms, which are significantly common in PC patients. By taking the required precautions and providing appropriate therapy, rational drug use targets can be reached.

**F for Feeding: **In PC patients, inadequate nutrition and, as a result, malnutrition are pretty prevalent. One of the most basic necessities for maintaining vital functions and improving quality of life is adequate and balanced nutrition. Therefore, nutrition plays a crucial role in enhancing the patient’s quality of life in PC. When the disease progresses, PC patients become malnourished for various reasons (severe anorexia, early satiety, chronic intestinal obstruction, nausea, vomiting, weakness, taste changes, etc.). Why are insufficient nutrition and malnutrition important? In malnourished patients, the musculoskeletal system, immune system, respiratory system, cardiovascular system, and nervous system are adversely affected due to which sarcopenia, the tendency to get infected, pressure sores, acute kidney failure, and increased mortality are observed. Nutritional care plays an essential role in symptom relief and as part of treatment, in addition to optimizing the quality of life and a sense of well-being.

Timing is important in determining the indication for PC and nutritional support in cancer patients. Parikh et al^33^ stated that nutritional support should be started simultaneously with curative treatments and in the early period. It takes a lot of time and effort to treat malnutrition and dietary deficiencies. Therefore, it is vital to provide adequate nutritional support at the right time. Nutrition, which is a dynamic process, is also a parametre that should not be ignored and remembered in the daily follow-up of patients. It should not be forgotten that the nutritional level of PC patients affects the quality of life, and appropriate nutritional support should be initiated by revealing the nutritional levels of the patients with nutritional screening tests^20,34^ (Table 4). The role of mnemonics is important in providing nutritional support with daily follow-ups.

**F amily Needs**: There is no doubt that the PC process is also a challenging period for sick family members. The quality of life of family members is directly related to the quality of life of the patient.

Family caregivers may become overwhelmed by the responsibilities placed on them. The structures of families are very diverse, and they may need psychosocial support.^35^ In the F part of the mnemonics, it is essential to evaluate the family needs and provide the necessary support. This need should also be taken into account and not forgotten as much as possible.

**E for Environment: **Palliative care patients with life-threatening diseases spend their final days in the hospital environment. In a way, this hospital environment, which has become their home, is the last resting place for most patients. This is a caring environment that will alleviate pain and suffering, and it is the key to improve one’s quality of life. Studies have revealed that the physical characteristics of the hospital and the socio-cultural relationship environment with the hospital staff can negatively affect the experiences of PC patients.

Sometimes even minor details can become important for patients with a life-threatening illness who spend their time in a hospital environment. These details are directly related not only to the physical environment but also to the social and cultural shares in the environment in which they live. In PC, in addition to focusing on treatment, pain relief, and other clinical interventions, providing an aesthetically pleasing and home-like warm environment not only increases their quality of life but also contributes to their recovery or to spend their last days in prosperity by preventing symptoms such as delirium-depression, agitation, and pain. Aesthetically pleasant and home-like environments have been demonstrated to improve the relaxation and positive emotional experiences of vulnerable patients in studies.^36,37^ Establishing empathy to create a suitable environment (sufficient daylight, suitable temperature level, prevention of noise, night sleep arrangement, proper timing of visitor hours, etc.) can be effective in providing a patient-specific environment and increasing communication power.

**H for Hospital Discharge (Preparation for Home Care): **No matter how hard PC professionals try to create a home-like environment for patients and family caregivers, patients desire to spend as much time as possible at home during their final days. Studies have shown that the majority of patients and family caregivers want to wait for death at home.^25,37^ It is critical to prepare the patient and caregivers for home care and provide the required training during PC follow-ups. Going home can be frightening, particularly for care providers. It is also part of the care process to help patients and their caregivers gain a sense of relief and confidence after psychological training. We implement this process with the training forms we prepare in our own clinic.

*Heading out of bed*, in addition, should be encouraged for the mobilization of patients whose general condition is suitable. Patients who are psychologically tired and fragile regard their bed as a castle and feel insecure outside of it. Fear of pain, anxiety that the disease may worsen, weakness, depression, and reluctance all limit the mobilization. The mobilization parametre should not be disregarded in PC patients whose overall state is suitable due to its psychological and physiological benefits.

**U for Ulcer (Skin Care): **Pressure ulcers are conditions associated with morbidity and mortality, causing both physical and psychosocial pain and distress in patients. Pressure ulcers are among the significant causes of mortality as a source of bleeding and infection.^38^ The number of deaths caused by pressure sores alone, as well as the cost of treatment, are far too large to be ignored. The best treatment for pressure ulcers, which both severely reduce the quality of life and have a high mortality rate, is to prevent their occurrence. After a pressure ulcer has formed, treatment is both more difficult and costly. Therefore, it is vital that pressure ulcers are followed closely, and wound care is given appropriately and regularly. Wound care with regular follow-ups is beneficial in preventing the development of pressure ulcers as well as stopping the progression of the ulcer’s stage and healing it. Awareness and mnemonics about pressure ulcers to provide a better quality of life can be efficient tools in preventing their occurrence.

**G for Gastrointestinal: **Gastrointestinal system (GIS) problems are frequently seen in PC due to existing diseases, multiple drug use, side effects of drugs used, and treatments such as chemotherapy and radiotherapy. Nausea-vomiting, diarrhoea, constipation, and cramps are all disturbing results that have a negative impact on one’s quality of life. Gastrointestinal system manifestations can aggravate pain and contribute to insomnia, fatigue, and weakness. This significantly impairs the quality of life of both the patient and caregivers. It is crucial that these disturbing and sometimes severe symptoms should be evaluated daily and given the necessary treatment and that they cannot be ignored for the quality of life of the patient and, therefore, the caregivers (Table 4).

**S for Social Support (Spiritual): **Palliative care patients are incredibly fragile and can easily lose their motivation. Therefore, social, emotional, and spiritual support becomes vital in this patient group. Psychosocial activities can help them feel better by improving communication, relaxation, awareness, and a sense of importance and utility. The need for spirituality grows as the process develops. Patients seek ways to deal with this process emotionally. Spirituality increases the patient’s sense of power and acceptance while reducing anxiety and depression rates. Spirituality has been shown to play a crucial role in coping with advanced diseases and enhancing patient well-being in studies. Spirituality and religion are different concepts. On the other hand, spirituality is defined as a notion that signifies the meaning of life, as well as a person’s attitude towards life, beyond religious belief and practice, and can gain new meaning as a result of the disease.^39,40^ People may have spiritual needs whether or not they follow a specific religion. Spiritual needs are an integral part of holistic care and are of great importance in terms of quality of life.

**I for Infection: **Due to a variety of disease-related and treatment-related factors, PC patients are particularly vulnerable to infections. Although there are contradictions in terms of antibiotic use in PC, they can be considered part of a good PC plan if life-threatening infections impair quality of life. The integration of PC with infection management is a novel approach that has the potential to reduce time-consuming procedures while also improving the service quality.^41^ Infection management becomes even more critical in the new patient profile (likely to recover) created by the evolution of PC.

**N for Need of Religion: **Each patient referred to PC comes from a different religious and cultural background. Thus, every patient or caregiver has (or does not) individualized beliefs and religiosity. Religiosity is related to the degree to which individuals believe in, follow, and practice a specific religion. When the heterogeneity of many societies is considered, it is seen that there are differences in approaches to PC and end-of-life problems in different cultures and religions. Decisions made are influenced by the beliefs of the patient and family caregivers.^39,42^ It is critical for end-of-life patients to contact a clergyman who is a member of their religion to minimize their anxiety and fear, strengthen their inner peace, and eliminate their loneliness. Sometimes, religious support can be more effective for patients and their relatives rather than treatment.

**P _3_ for Palliative (P**hysiotherapy, **P**sychotherapy, **P**repar­edness): *Physiotherapy* strives to maximize movement and function to improve movement potential and treatment and rehabilitation to achieve a higher quality of life. On a patient-centred basis, physiotherapists are part of a multidisciplinary PC team to improve patients’ quality of life who are regarded to be in need. It is suggested that patients with the most frequent symptoms requiring PC, such as pain, weakness, cough, and shortness of breath, can benefit from symptom control with physical therapy.^43^ In addition to physical activity, psychological activation and relaxation are also highly beneficial in PC patients.

*Psychotherapy* is a psychological intervention that can assist patients and caregivers in coping with the process while also reducing anxiety, fear, and pain. Studies have proposed that meaning-centred psychotherapy can help to cope with difficulties.^44,45^

*Preparedness* for care has both a physical and emotional component. Family caregivers’ preparedness for physical care, challenges, and death of the patient can be regarded as a protective factor against adverse outcomes.^46^

**C for **Goals of **C**are** (Communication): **Care goals include planning treatments, the intensity of care, planning future care needs, determining what is essential to the patient, resuscitation preferences, and discussing prognosis. The care goals of the patients should be aligned with their preferences and values and planned early.

Communication is a must-have tool in setting care goals. It is crucial in assisting the patient in accepting reality and adapting to challenges.

## Conclusion

Palliative care is a type of care that needs to be performed with the help of multidisciplinary teams. Some details that are significant to the patient and family caregivers may be forgotten or missed while the focus is on treatment. However, with this mnemonic, essential interventions can be made, and teams such as nurses, physiotherapists, psychotherapists, clergy, and nutrition teams can readily be incorporated into the care. We anticipate that all relevant multidisciplinary teams will be engaged in care by using this simple mnemonic, resulting in a considerable improvement in quality of life.

## Figures and Tables

**Table 1.** Parametres of SAFE HUGS IN PC

**Initial**	**Main Title**	**Subheadings**	
**S**	**S**LEEP PATTERNS	**S**leep disturbances **S**edatives	
**A 5**	**A**NALGESIA	**A**nalgesia *(pain control)* **A**gitation **A**nxiety *(Antidepresants-Anticonvulsant)*	*5 A components*
**F**	**F**EEDING	**F**eeding *(nutritional support)* **F**amily needs	
**E**	**E**NVIRONMENT	**E**nvironment *(enough daylight, noise, visitors staying late,temperature control, suitable environment to prevent delirium)* **E**mpathy	
**H**	**H**OSPITAL DISCHARGE HOME CARE	**H**ospital bed avoidance *(discharge from* *hospital)* **H**eading out of bed *(mobilization)*	
**U**	**U**LCER	**U**lcer; pressure **u**lcer-skin care	
**G**	**G**ASTROINTESTINAL	Constipation, nausea-emesis, diarrhoea	
**S**	**S**OCIAL	**S**ocial motivation **S**piritual and emotional **s**upport	
**I**	**I**NFECTION	**I**nfection, inflammation	
**N**	**N**EED OF RELIGIOUS	**N**eed of religious; support in accordance with his religious belief	
**P 3**	**P**ALLIATIVE	**P**hysiotherapy **P**sychotherapy **P**reparedness	*3 P components*
**C**	**C**ARE	**C**onsultation Goals of **c**are Communication	

**Table 2.** Pharmacologic Management for Sleep Disturbances^18,20^

**Sedative-Hypnotics**	General Doses*	
Zolpidem	5-20 mg	Lower dose should be used for older or debilitated individuals, patients with impaired hepatic function
Zaleplon	5-20 mg	Lower dose should be used for older or debilitated individuals, patients with impaired hepatic function, and patients taking cimetidine
Eszopiclone	1-3 mg	Lower dose should be used for older or debilitated individuals
**Benzodiazepines**		
Flurazepam	15-30 mg	Lower dose should be used for older or debilitated individuals
Estazolam	0.5-2 mg	Lower dose should be used for older or debilitated individuals
Temazepam	7.5-30 mg	Lower dose should be used for older or debilitated individuals
Triazolam	0.125-0.25 mg	Lower dose should be used for older or debilitated individuals
Quazepam	7.5-15 mg	Actual doses should be determined on an individual basis
**Melatonin receptor agonists**		
Ramelteon	8 mg	Approved for long-term use
**Antidepressants**		
Doxepin	3-6 mg	-
Amitriptyline	10-100 mg	-

^*^Actual doses should be determined on an individual basis.

**Table 3.** Pain Management^20,24,27-29^

**Opioids for Pain Management (Adult)**		
	**Starting Oral Doses a**	**Duration of Action**
Morphine	15-30 mg	3-6 hours
Codeine	15-60 mg	4-6 hours
Hydromorphone	2-4 mg	4-5 hours
Hydrocodone	2.5-10 mg	4-8 hours
Oxycodone	5-10 mg	3-4 hours
Oxymorphone	10 mg	8-12 hours
Methadone	5-10 mg	4-6 hours
Fentanyl (buccal tablet)	100-200 μg	2-4 hours
Fentanyl (transdermal patch)	25 μg h^-1^(worn for 3 days)	48-72 hours
Buprenorphine (transdermal patch)	5-10 μg h-1 (worn for 7 days)	-
**Oral Adjuvant Analgesics**		
	**Typical Starting Dose**	**Usual Effective Dose**
Gabapentin	100-300 mg once daily	300-1200 mg twice or thrice daily
Pregabalin	25-75 mg twice daily	300-600 mg twice daily
Carbamazepine	50-100 mg twice daily	300-800 mg twice daily
Topiramate	25-50 mg daily	50-200 mg twice daily
Oxcarbazepine	75-150 mg twice daily	150-800 mg twice daily
Tiagabine	4 mg a	4-12 mg twice daily
Tricyclic antidepressants Amitriptyline Nortriptyline Desipramine	10-25 mg	50-150 mg
Dexamethasone	1-2 mg	-

^a^Doses given are guidelines for opioid-naïve patients; actual doses should be determined on an individual basis.

**Table 4.** Management of Nutrition and Gastrointestinal System Problems

**Nutrition Management (Adult)**		
Energy requirement	25-30 kcal kg^-1^ day^-1^	Should be measured individually
Protein requirement	1-1.5 g kg^-1^ day^-1^	Should be measured individually
Choice of energy substrates	Increase the ratio of energy from fat	Should be measured individually
**Pharmacologic Management of Nausea and Vomiting**		
	**Typical Starting Dose **	**Frequency**
Metoclopramide	10-20 mg PO IV^-1^ SC^-1^	Every 6-8 hours
Ondansetron	4-8 mg PO IV^-1^	1 or 2 times daily
Granisetron	1 mg	Twice daily
Mirtazapine	15-45 mg PO	Every night
Promethazine	25 mg PO	4-6 hours
**Constipation**		
**First-Line Treatment**	**Second-Line Treatment**	**Evaluate Possible Causes**
Oral laxatives	Rectal suppository and enema	Exclude intestinal obstruction and opioid-induced constipation

PO, Per Oral; IV, intravenously; SC, subcutaneously.

General comments and recommendations of cancer patients. It must be determined on an individual basis.

## References

[1] Demir M Palliative care ethics. Yoğun Bakim Derg. 2016;7(2):62 66. 10.5152/dcbybd.2016.1202)

[2] Ferris FD Librach SL . Models, standards, guidelines [guidelines]. Clin Geriatr Med. 2005;21(1):17 44. 10.1016/j.cger.2004.08.002) 15639035

[3] Hawley PH The bow tie model of 21st century palliative care. J Pain Symptom Manag. 2014;47(1) :e2 e5. 10.1016/j.jpainsymman.2013.10.009) 24321509

[4] Ballou JH Brasel KJ . Palliative care and geriatric surgery. Clin Geriatr Med. 2019;35(1):35 44. 10.1016/j.cger.2018.08.004) 30390982

[5] Vincent JL Give your patient a fast hug (at least) once a day. Crit Care Med. 2005;33(6):1225 1229. 10.1097/01.ccm.0000165962.16682.46) 15942334

[6] Vincent WR 3rd Hatton KW . Critically ill patients need “FAST HUGS BID” (an updated mnemonic). Crit Care Med. 2009;37(7):2326 2327. 10.1097/CCM.0b013e3181aabc29) 19535943

[7] Nair AS Naik VM Rayani BK . FAST HUGS BID: modified mnemonic for surgical patient. Indian J Crit Care Med. 2017;21(10):713 714. 10.4103/ijccm.IJCCM_289_17) 29142387 PMC5672681

[8] Şenoğlu N Köse I Zincircioğlu Ç Erbay RH . Fast hugs with Intensive Care Unit. tybdd. 2014;12(3):72 81. 10.4274/tybdd.69885)

[9] Berger AM Update on the state of the science: sleep-wake disturbances in adult patients with cancer. Oncol Nurs Forum. 2009;36(4):E165 E177. 10.1188/09.ONF.E165-E177) 19581220

[10] Berger AM Parker KP Young-McCaughan S et al. Sleep wake disturbances in people with cancer and their caregiver: state of science. Oncol Nurs Forum. 2005;32(6):E98 E126. 10.1188/05.ONF.E98-E126) 16270104

[11] Pawl JD Lee SY Clark PC Sherwood PR . Sleep characteristics of family caregivers of individuals with a primary malignant brain tumor. Oncol Nurs Forum. 2013;40(2):171 179. 10.1188/13.ONF.171-179) 23448742 PMC3880563

[12] Lee KC Hsieh YL Lin PC Lin YP . Sleep pattern and predictors of sleep disturbance among family caregivers of terminal ill patients With cancer in Taiwan: a longitudinal study. Am J Hosp Palliat Care. 2018;35(8):1109 1117. 10.1177/1049909118755453) 29390869

[13] Slocum-Gori S Hemsworth D Chan WW Carson A Kazanjian A . Understanding compassion satisfaction, compassion fatigue and burnout: a survey of the hospice palliative care workforce. Palliat Med. 2013;27(2):172 178. 10.1177/0269216311431311) 22179596

[14] Hlubocky FJ Sher T Cella D et al. The impact of sleep disturbances (SD) on quality of life, psychological morbidity, and survival of advanced cancer patients (ACP) and caregivers (CG). J Clin Oncol. 2017;35(15_suppl):10115. 10.1200/JCO.2017.35.15_suppl.10115)

[15] Carter PA A brief behavioral sleep intervention for family caregivers of persons with cancer. Cancer Nurs. 2006;29(2):95 103. 10.1097/00002820-200603000-00003) 16565618

[16] Feng F Zhang Y Hou J et al. Can music improve sleep quality in adults with primary insomnia? A systematic review and ­network meta-analysis. Int J Nurs Stud. 2018;77:189 196. 10.1016/j.ijnurstu.2017.10.011) 29100201

[17] Valero-Cantero I Carrión-Velasco Y Casals C Martínez-Valero FJ Barón-López FJ Vázquez-Sánchez MÁ . Intervention to improve quality of sleep of palliative patient carers in the community: protocol for a multicentre randomised controlled trial. BMC Nurs. 2020;19(1):107. 10.1186/s12912-020-00501-2) PMC7670676 33292183

[18] Schutte-Rodin S Broch L Buysse D Dorsey C Sateia M . Clinical guideline for the evaluation and management of chronic insomnia in adults. J Clin Sleep Med. 2008;4(5):487 504. 10.5664/jcsm.27286) 18853708 PMC2576317

[19] Tariq SH Pulisetty S . Pharmacotherapy for insomnia. Clin Geriatr Med. 2008;24(1):93 105. 10.1016/j.cger.2007.08.009) 18035234

[20] National Comprehensive Cancer Network. NCCN Clinical ­Practice Guidelines in Oncology. Availableat. Available at: https://www.nccn.org/professionals/physician_gls/default.aspx. Last Accessed October 8, 2018.

[21] American Pain Foundation. Breakthrough cancer pain: mending the break in the continuum of care. J Pain Palliat Care Pharmacother. 2011;25(3):252 264. 10.3109/15360288.2011.599920) 21882979

[22] Gao W Gulliford M Higginson IJ . Prescription patterns of analgesics in the last 3 months of life: a retrospective analysis of 10,202 lung cancer patients. Br J Cancer. 2011;104(11):1704 1710. 10.1016/j.pop.2011.03.004) 21540860 PMC3111163

[23] Breuer B Fleishman SB Cruciani RA Portenoy RK . Medical oncologists’ attitudes and practice in cancer pain management: a national survey. J Clin Oncol. 2011;29(36):4769 4775. 10.1200/JCO.2011.35.0561) 22084372

[24] Dalal S Bruera E . Assessment and management of pain in the terminally ill. Prim Care. 2011;38(2):195 223. 10.1016/j.pop.2011.03.004) 21628035

[25] Sucaklı MH Koşar Y . Palliative care and quality of life. Klin Tıp Aile Hekimliği Derg. 2016;8(3):34 39.

[26] Davidson’s Principles and Practice of Medicine. Colledge, Stuart Ralston, Ian D. Penman Churchill Livingstone/Elsevier, 2014:283 292. ISBN 0702050474, 9780702050473.

[27] United States Food and Drug Administration. Drug approvals and database. Available at: https://www.fda.gov/Drugs/InformationOnDrugs/default.htm. Accessed October 8, 2018.

[28] Swetz KM Kamal AH . In the clinic. Palliative care. Ann Intern Med. 2012;156(3). 10.7326/0003-4819-156-3-201202070-01002) 22312158

[29] Argoff CE Silvershein DI . A comparison of long- and short-acting opioids for the treatment of chronic noncancer pain: tailoring therapy to meet patient needs. Mayo Clin Proc. 2009;84(7):602 612. 10.1016/S0025-6196(11)60749-0) 19567714 PMC2704132

[30] Dolinsky C Metz JM . Palliative radiation therapy in oncology. Anesthesiol Clin. 2006;24(1):113 128. 10.1016/j.atc.2005.11.002) 16487898

[31] Qaseem A Snow V Shekelle P et al. Evidence-based interventions to improve the palliative care of pain, dyspnea, and depression at the end of life: a clinical practice guideline from the American College of Physicians. Ann Intern Med. 2008;148(2):141 146. 10.7326/0003-4819-148-2-200801150-00009) 18195338

[32] Stoklosa J Patterson K Rosielle D Arnold RM . Anxiety in palliative care: causes and diagnosis #186. J Palliat Med. 2011;14(10):1173 1174. 10.1089/jpm.2011.9644) 22004149

[33] Parikh RB Kirch RA Smith TJ Temel JS . Early specialty palliative care—translating data in oncology into practice. N Engl J Med. 2013;369(24):2347 2351. 10.1056/NEJMsb1305469) 24328469 PMC3991113

[34] Arends J Bachmann P Baracos V et al. ESPEN guidelines on nutrition in cancer patients. Clin Nutr. 2017;36(1):11 48. 10.1016/j.clnu.2016.07.015) 27637832

[35] National Consensus Project for Quality Palliative Care Consortium Organizations. Clinical Practice Guidelines for Quality Palliative Care. 4th ed. Pittsburgh, PA: National Consensus Project; 2018.

[36] Robinson J Gott M Gardiner C Ingleton C . The impact of the environment on patient experiences of hospital admissions in palliative care. BMJ Support Palliat Care. 2018;8(4):485 492. 10.1136/bmjspcare-2015-000891) 26408427

[37] Timmermann C Uhrenfeldt L Høybye MT Birkelund R . A palliative environment: caring for seriously ill hospitalized patients. Palliat Support Care. 2015;13(2):201 209. 10.1017/S147895151300117X) 24524598

[38] Ferris A Price A Harding K . Pressure ulcers in patients receiving palliative care: a systematic review. Palliat Med. 2019;33(7):770 782. 10.1177/0269216319846023) 31018829

[39] Evangelista CB Lopes ME Costa SF Batista PS Batista JB Oliveira AM . Palliative care and spirituality: an integrative literature review. Rev Bras Enferm. 2016;69(3):591 601. 10.1590/0034-7167.2016690324i) 27355311

[40] Selman L Harding R Gysels M Speck P Higginson IJ . The measurement of spirituality in palliative care and the content of tools validated cross-culturally: a systematic review. J Pain Symptom Manage. 2011;41(4):728 753. 10.1016/j.jpainsymman.2010.06.023) 21306866

[41] Stone PW Agarwal M Ye F et al. Integration of palliative care and infection management at the end of life in U.S. Nurs Homes J Pain Symptom Manag. 2019;58(3):408-416.e1. 10.1016/j.jpainsymman.2019.06.001) PMC6708746 31195078

[42] Steinberg SM Cultural and religious aspects of palliative care. Int J Crit Illn Inj Sci. 2011;1(2):154 156. 10.4103/2229-5151.84804) 22229141 PMC3249849

[43] Kumar SP Jim A . Physical therapy in palliative care: from symptom control to quality of life: a critical review. Indian J Palliat Care. 2010;16(3):138 146. 10.4103/0973-1075.73670) 21218003 PMC3012236

[44] Rosenfeld B Saracino R Tobias K et al. Adapting Meaning-Centered Psychotherapy for the palliative care setting: results of a pilot study. Palliat Med. 2017;31(2):140 146. 10.1177/0269216316651570) 27435603 PMC5473503

[45] Strada E Sourkes A Barbara M . Psychotherapy in the palliative care setting. Prim Psychiatry. 2009;16(5):34 40.

[46] Alvariza A Häger-Tibell L Holm M Steineck G Kreicbergs U . Increasing preparedness for caregiving and death in family caregivers of patients with severe illness who are cared for at home - study protocol for a web-based intervention. BMC Palliat Care. 2020;19(1):33. 10.1186/s12904-020-0530-6) PMC7079472 32183803

[47] Dunlay SM Strand JJ . How to discuss goals of care with patients. Trends Cardiovasc Med. 2016;26(1):36 43. 10.1016/j.tcm.2015.03.018) 25933831 PMC4592692

